# The Remote Assessment and Dynamic Response Program: Development of an In-Home Dementia-Related Care Needs Assessment to Improve Well-Being

**DOI:** 10.1093/geroni/igac006

**Published:** 2022-02-07

**Authors:** Lyndsey M Miller, Diane N Solomon, Carol J Whitlatch, Shirin O Hiatt, Chao-Yi Wu, Christina Reynolds, Wan-Tai Michael Au-Yeung, Jeffrey Kaye, Joel S Steele

**Affiliations:** 1 School of Nursing, Oregon Health and Science University, Portland, Oregon, USA; 2 Oregon Center for Aging & Technology (ORCATECH), Oregon Health and Science University, Portland, Oregon, USA; 3 Benjamin Rose Institute on Aging, Cleveland, Ohio, USA; 4 NIA-Layton Aging & Alzheimer’s Disease Research Center, Oregon Health and Science University, Portland, Oregon, USA; 5 Department of Psychology, Portland State University, Portland, Oregon, USA

**Keywords:** Care values, Digital activity data, Dyadic intervention, Technology, Unmet needs

## Abstract

**Background and Objectives:**

The Remote Assessment and Dynamic Response (READyR) Program was developed in order to address the current lack of early-stage dementia care planning programs that assess the care needs of persons with dementia. The goal was to create a program informed by care values and ongoing ecologically valid data. The objectives of this study are to describe the development and design process of the READyR Program, and to evaluate the utility of the READyR Program for identifying dementia-related care needs.

**Research Design and Methods:**

A prototype of the web-based READyR Program tool was first created using digital activity data that were collected by previous studies using a platform of multimodal sensors installed in the homes of older adult couples with and without dementia. Digital activity data were then mapped onto potential care values (e.g., safety & autonomy) to create a values-based needs assessment that is tailored to the individual care dyad. Next, evaluation of the READyR Program by 11 professional dementia care coordinators and case managers (across 3 semistructured focus groups) was used to explore the utility of READyR for assessing dementia-related needs. Qualitative description using conventional content analysis was used to iteratively code focus group data and to describe prevalent themes.

**Results:**

Prevalent focus groups themes included barriers to (e.g., family relationship strain) and facilitators of (e.g., tailored assessments) the optimal process for assessing dementia-related care needs by care coordinators, as well as advantages to (e.g., providing new objective insights into function, and routines) and disadvantages of (e.g., bringing up new questions about care) incorporating the remote monitoring data into a values-based needs assessment.

**Discussion and Implications:**

READyR has the potential to help family members, as well as care coordinators and providers, gain insight into the values-based care needs of persons with early-stage dementia.

**Clinical Trials Registration Number:** NCT04542109

**Translational Significance:** This study addresses the question: Can persons living with dementia and their spouses or other family care partners plan for dementia-related care needs and improve both persons’ well-being through remote assessment and in-home passive sensing technology? The Remote Assessment and Dynamic Response (READyR) Program was developed using digital activity data from the homes of older adults and found to be useful for a dementia care needs assessment according to focus group feedback from care coordinators. READyR has the potential to provide a more continuous and ecologically valid assessment of changes in daily routines and the related dementia care needs indicated.

Despite advancements in the diagnosis of Alzheimer’s disease and related dementias, there has been little progress in the standardization of assessment for everyday dementia-related care needs in the home. Clinical assessment of dementia emphasizes cognitive testing, which gives little insight into unmet needs related to functional deficits or the care environment. As a result, unmet dementia-related care needs are highly prevalent, and threaten the well-being, safety, and ability to age in place for persons living with dementia and also for their spouses or other family care partners (Black et al., 2013, 2019; Boots et al., 2015; De Poli et al., 2020; Gaugler et al., 2005; Monin et al., 2019; NASEM, 2021). Two major components of a dementia-related care needs assessment are notably absent from both standard practice and from evidence-based support programs. First, few begin with an assessment of the care values of the person living with dementia (Whitlatch et al., 2017), which is critical to achieving person-centered care (Fazio et al., 2018). Second, there is no standard, objective method for collecting ongoing in-home assessments of the routines and activities that make up the everyday care situation (De Poli et al., 2020), which is essential to understanding care needs as dementia advances. The Remote Assessment and Dynamic Response (READyR) Program was developed in order to address the current lack of dementia care planning programs that assess the care needs through: (a) knowledge of the care values of the person living with dementia, and (b) ongoing ecologically valid data from the home setting.

## Values-Based Care Planning

Effective dementia care requires planning that is tailored to the care values and specific needs of the person living with dementia, especially in the early stage of dementia when they can and should be more involved (De Poli et al., 2020; Monin et al., 2019; Whitlatch & Orsulic-Jeras, 2018). In a recent consensus study report on caring for persons living with dementia, the National Academies of Science, Engineering, and Medicine identified “attention to each person’s needs and values” as a guiding principle for dementia care (NASEM, 2021). The READyR Program considers needs and values to be inextricable. Assessing the care values of persons living with dementia is an essential first step to determining care needs, because a need can only be perceived if the current care situation does not uphold their values (e.g., highly valuing autonomy but having little independence in daily routines). This perspective is in line with theoretical models (Lord et al., 2020) and other evidence-based dementia care planning interventions, such as the SHARE (Support, Health, Activities, Resources, and Education) Program (Whitlatch et al., 2017) and the group-based EPIC (Early-Stage Partners in Care) Program (Coon et al., 2017), both of which help families identify, accept, and act on a person’s values and needs for care. The READyR Program is distinct from other dementia care planning programs in that it specifically targets the match between the care values and daily routines of the person living with dementia.

## Assessment of Daily Routines

The ability to identify dementia-related care needs is currently limited by reliance upon assessments in clinical settings, which is problematic for two reasons. First, assessment in the clinical setting lacks ecological validity. Observing everyday function, routines, and safety issues must be done within the home environment to gain a realistic assessment. Second, relying upon self-reports from persons living with dementia and their care partners to determine care needs, which is common practice in the clinical setting, can be inaccurate or incomplete. The unreliability of self-reports is exacerbated by impaired insight and executive dysfunction among persons living with dementia, and by stress among care partners, which can affect their appraisal of the disease progression (Godefroy et al., 2014; Orfei et al., 2010; Wild et al., 2016). In-home assessments conducted by a care coordinator or home health care team, although not standard practice, are ideal for gaining a baseline window into the full care environment. Assessing for safety (e.g., loose rugs, poor lighting, assistive devices, safe access) and observing how routines are completed within the home is both ecologically valid and objective. However, due to cost and scarcity of resources, these in-home assessments are not usually completed frequently enough to detect changes in care needs, which are certain to come in dementia, and in the brief observation window in which they are conducted it is difficult to gain a full and naturalistic picture of the in-home activity of the family.

## Remote Assessments

A promising solution to many of these assessment challenges is remote in-home sensing or remote activity monitoring, which can provide objective and continuous monitoring and ecologically valid assessments (Gaugler et al., 2018; Thomas et al., 2020). Remote sensing systems have been designed for a variety of purposes, and are becoming widely accepted, even covered by Medicaid (Berridge, 2018). Passive remote monitoring systems for older adults’ homes collect data in order to inform longitudinal research on activity and function, or response to treatment (Beattie et al., 2020; Block et al., 2016; Kabelac et al., 2019; Thomas et al., 2020), and can be adapted to inform care providers or other individuals supporting care about early warning signs of safety issues or health declines (Skubic et al., 2015; Wild et al., 2021). Remote sensing systems can also be designed as dynamic and interactive to support older adults and persons with dementia at home (Consel et al., 2017).

Other dementia care management interventions have been conducted remotely via telephone and internet and shown efficacy (Bass et al., 2019; Possin et al., 2019). Yet, conducting a needs assessment by incorporating wearables, passive sensors, and other methods of remote monitoring has been challenging for a variety of reasons. Some of the barriers that have prevented remote home-based assessment from becoming more ubiquitous and integrated within dementia care interventions include: a lack of standardized approaches, high cost, team science requirements, proprietary algorithms and other access-limiting intellectual property rights (Beattie et al., 2020), and assumptions about lack of feasibility and poor uptake of technology by older adults (Mannheim et al., 2019). Despite these barriers, a minimally obtrusive digital health-enabled study system has been developed by the Oregon Center for Aging and Technology (ORCATECH), and through a large interagency funding effort (National Institutes of Health and the Department of Veterans Affairs) has recently been demonstrated to be capable of monitoring multiple domains of health and wellness among diverse older adults with and without cognitive impairment in a range of environments (Beattie et al., 2020).

## The READyR Framework

**Figure 1.**
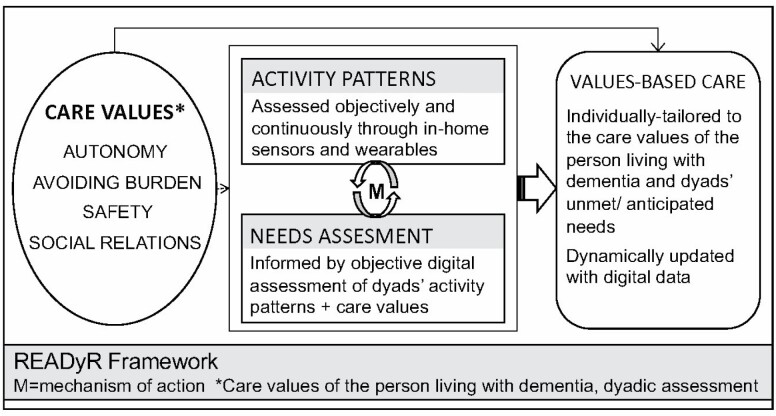
The Remote Assessment and Dynamic Response (READyR) Program framework.

In developing READyR, the overall goal was to create a dynamically tailored program that has the potential to inform current and future care needs, mitigate the stress process, and improve health and well-being in the care dyad as dementia progresses. The conceptual model for READyR (see Figure 1) hinges upon an understanding of the care values of the person living with dementia. The hypothesized mechanism of action in achieving values-based care is identifying unmet needs based upon the incongruence between those care values and the realities of dyads’ daily life that can be observed by digital activity patterns. The Stress Process Model (Judge et al., 2010; Pearlin et al., 1990) and our previous work applying this model to studies of care values in dementia care dyads (Miller et al., 2018, 2019) provide the theoretical basis for understanding the process by which unmet or overly stressful care needs become detrimental to the well-being of the dementia care dyad (person living with dementia and a family care partner).

According to the Stress Process Model, activity patterns such as increased dependency in activities of daily living begin as stressors that can be objectively observed. These objective stressors then contribute to secondary subjective strains from an unmet need (e.g., social isolation and relationship strain in the dyad), leading to further negative outcomes such as poorer physical and mental health, and diminished quality of life (Judge et al., 2010; Pearlin et al., 1990). A critical step to implementing a plan that mitigates the stress process before it proliferates to subjective strains is thus understanding objective stressors by gaining insight into the dyad’s in-home activity patterns and the impending care needs of the person with dementia.

The objectives of this paper are:

1. To describe the development and design process of the READyR Program
2. To evaluate the utility of the READyR Program for identifying dementia-related care needs based on results from focus group testing with dementia care coordinators

## Research Design and Methods

### READyR Development

**Figure 2.**
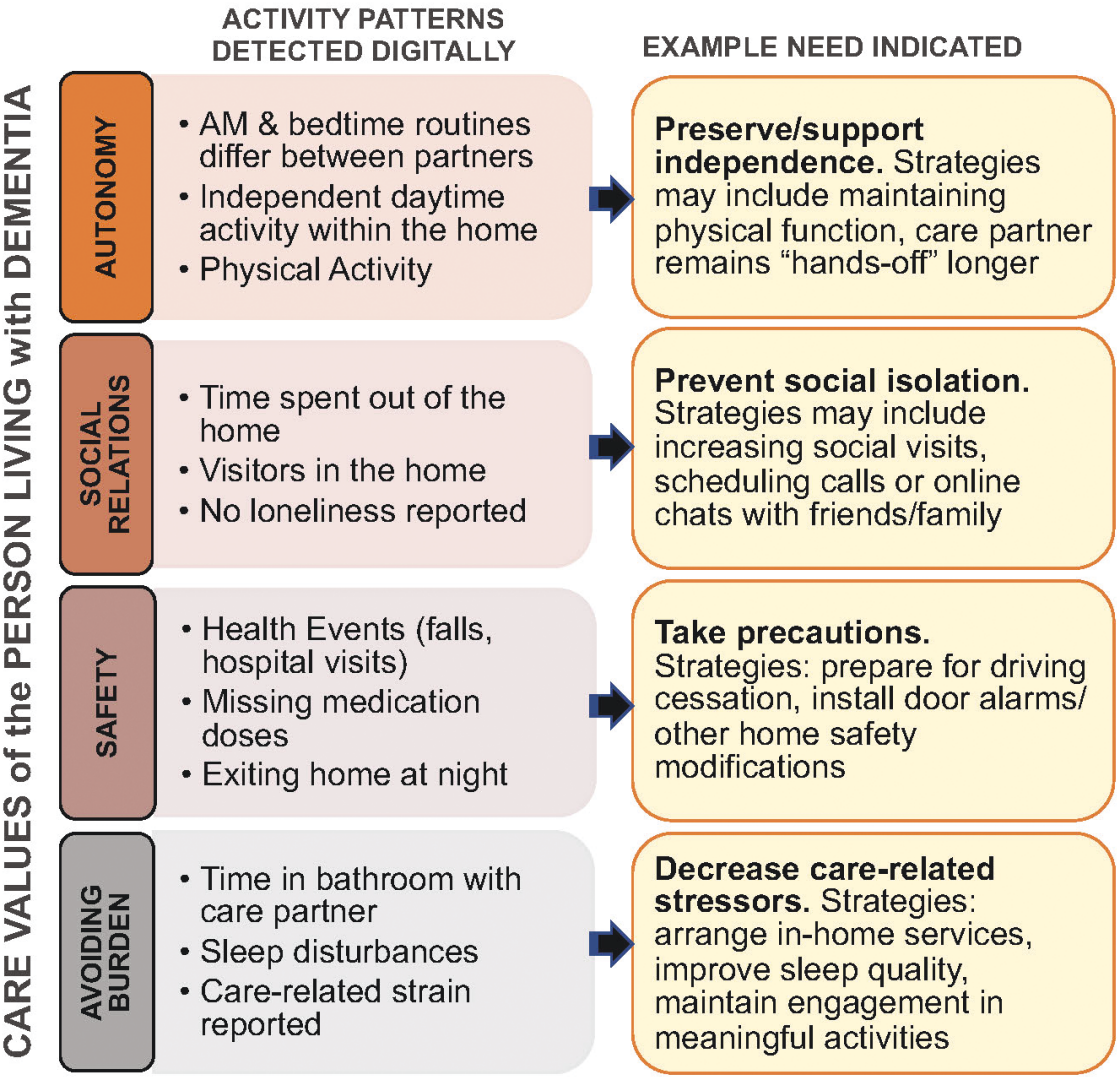
Care-related needs assessed through care values and digital activity patterns.

The READyR Program involves conventional clinical and subjective assessments, as well as the implementation of a previously developed in-home sensor platform. The ORCATECH sensor platform was used as the technological starting point in order to objectively assess daily activity patterns of persons living with dementia and their spouses using continuous ecological assessments and weekly online surveys. The ORCATECH sensor platform is described in-depth elsewhere (Beattie et al., 2020; Thomas et al., 2021). Briefly, the technology front end includes passive infrared sensors that capture home exits and activity within/transitions between rooms, an actigraphy watch detects step count and sleep duration, an electronic pillbox tracks medication-taking behavior, a scale records daily weight, and an under-the-mattress bedmat captures a variety of sleep metrics (e.g., bed exits at night, total sleep time) and physiologic data such as heart rate and respirations. The READyR Program uses an initial 3 months of sensor data to establish in-home activity patterns (Bernstein et al., 2021). Activity patterns are then matched with ratings from both dyad members on the importance of four care values to the dyad member who is living with dementia: autonomy, safety, social relations, and avoiding burden (Figure 2).

READyR is designed so that the person living with dementia and care partner both rate the care values (of the person living with dementia) in the baseline assessment. Then, in the initial READyR intervention session the activity data are reviewed with the care dyad in order to determine how well they feel that they are supporting the care values according to their typical routines. For example, if autonomy is very important to the person living with dementia, but it appears that the dyad has synchronous nighttime and morning routines and neither person leaves the house alone, it may indicate less than optimal autonomy. In the second READyR session, individual dyads will set goals for daily patterns to stabilize or align their activity with their values in order to meet their current and future care needs. Then, in a follow-up period of 5 months, goals can be dynamically adjusted as needed over time, when sensor activity patterns indicate a change in activity patterns and routines.

**Figure 3.**
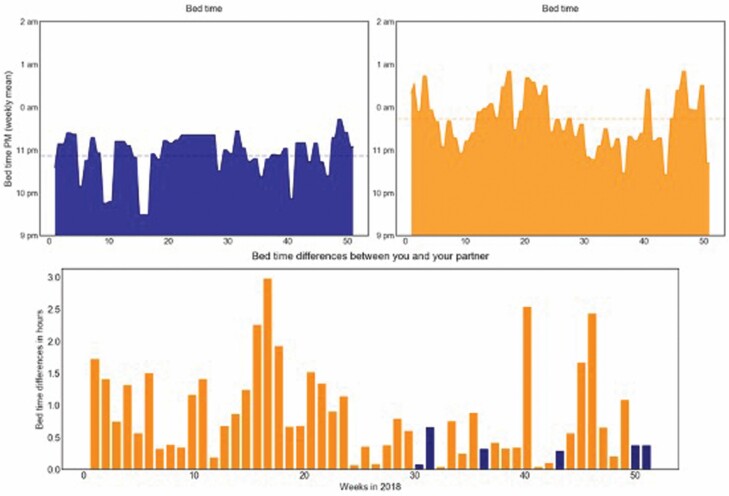
Asynchronous daily routines between the person living with dementia (in blue) and their spouse (in orange; pertaining to the care value of autonomy).

**Figure 4.**
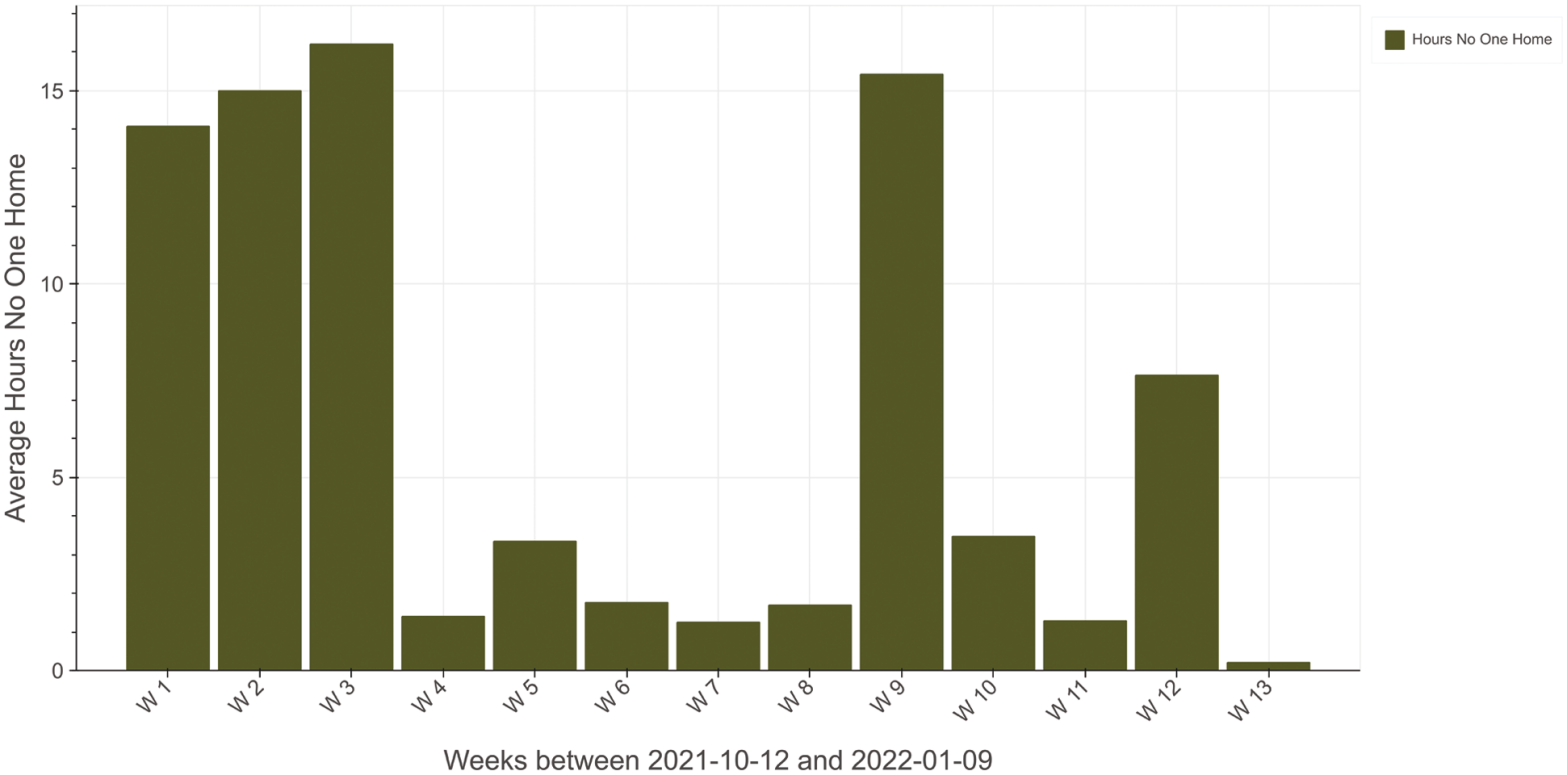
Time out of home (pertaining to the care value of social relations).

**Figure 5.**
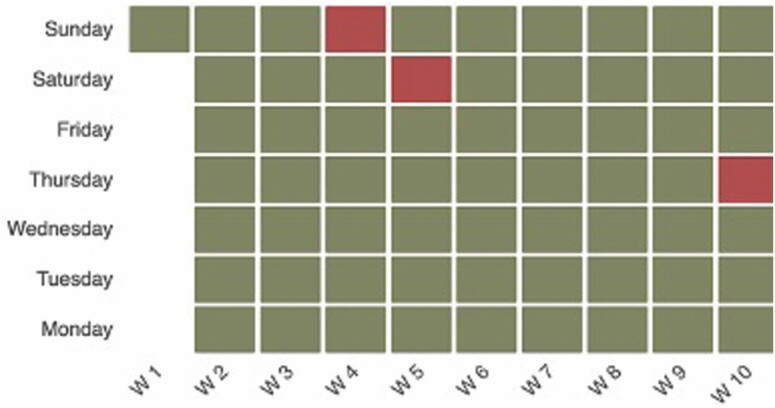
Electronic pill box data: days in red indicate pill box not opened (pertaining to the care value of safety).

**Figure 6.**
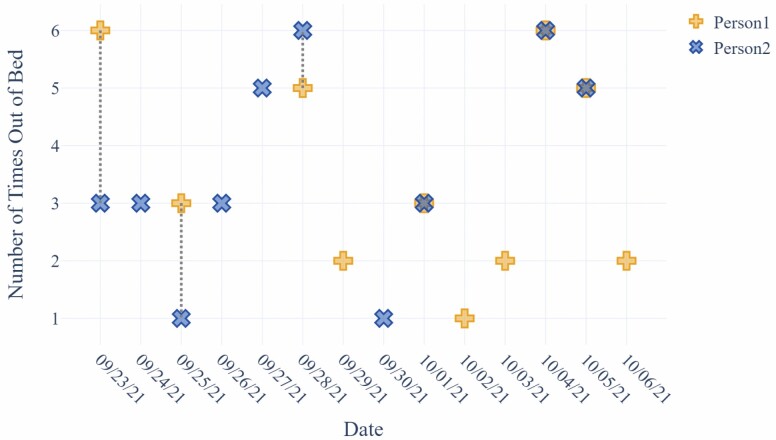
Sleep disturbances (number of bed exits at night) for the person living with dementia (in blue) and their spouse (in yellow; pertaining to the care value of avoiding burden on one’s family).

To help participants visualize their own data in intervention sessions, a tailored web-based tool was created. Data specific to each dyad’s home were pulled from the ORCATECH central server via Python. These data were processed and analyzed in Python to generate plots and figures of activity related to participants’ care values. Plots and figures were incorporated into an HTML template to create a static web page that reflected participant activity for specific time periods (examples in Figures 3–6). Data visualization principles were applied by the READyR study investigators and data specialists, and the web-based tool was created through an iterative process with the study team and in focus groups described below.

### Focus Group Sample and Setting

To explore the utility of READyR, expert feedback was solicited through focus groups with dementia care coordinators who have experience assessing care needs of persons living with dementia and their care partners. Research was conducted through a major health sciences university in the Northwestern United States, and ethical approval received through the university’s Institutional Review Board. Purposive sampling (Creswell, 2012) for three focus groups was used to recruit care professionals with extensive experience with geriatrics and dementia care coordination. Focus group participants were recruited from local organizations and institutions providing care coordination services to persons living with dementia and their families. Recruitment methods included a flyer sent by e-mail to outpatient clinics, stand-alone care management services, and included in local research presentations. Follow-up phone calls were made by the research team to screen potential participants for the eligibility criteria. Interested participants were included if they were at least 21 years old, involved in guiding care planning for older adults who have been diagnosed with Alzheimer’s disease or a related dementia (any stage of disease), and able and willing to attend a focus group by videoconferencing. Participants received a $100 gift card for their time and participation in a single focus group lasting approximately 90 min.

### Focus Group Procedures and Analysis

Three focus groups were facilitated by a psychiatric nurse practitioner with extensive training in group dynamics and delicate conversations. Focus groups were conducted over secure videoconference during the fall of 2020 (during University restrictions against in-person research due to the coronavirus disease 2019 pandemic). The groups lasted from 60 to 90 min and were steered by a semistructured interview guide (see Supplementary Appendix). To gain an unbiased view of the potential added value of the READyR Program, the interview guide explored professionals’ current assessment practices in detail prior to introducing any information on the READyR Program. Subsequently, a brief case study and a scripted slideshow were shown to participants to familiarize them with digital activity data from a care dyad’s home. These data explained sleep patterns, physical activity, medication-taking behavior, and home activity for both dyad members. Focus group participants were asked to reflect on what these remote digital data might add to their current assessment practices, and also how these data might inform their understanding of the degree to which the dyad is able to uphold potential care values of autonomy, avoiding being a burden on family, safety, and social relations (see Supplementary Appendix for semistructured focus group interview guide).

Focus groups were recorded during videoconferences and digitally transcribed verbatim. Prior to transcription, the primary qualitative expert reviewed each focus group recording twice and completed a field note summarizing and reflecting on each focus group (Phillippi & Lauderdale, 2018). Following transcription, a qualitative expert (D. N. Solomon), a research associate (S. O. Hiatt), and the study’s principal investigator (L. M. Miller) employed qualitative description (QD; Sandelowski, 2010) using conventional content analysis (Hsieh & Shannon, 2005). QD has been recognized as an ideal method to describe the “who, what, and where” of experiences (Kim et al., 2017), and for its flexibility in sampling and data collection (Colorafi & Evans, 2016). In imparting knowledge about persons with dementia, QD also offers a method for accessing firsthand knowledge and experiences of experts in the field (Neergaard et al., 2009). QD derives from the interpretive turn (Denzin & Lincoln, 2005), and is subjective and naturalistic in seeking participant’s views of a phenomena (Bradshaw et al., 2017). In its descriptive rigor, QD is also ideally suited to scaffold upon, informing further mixed methods or quantitative research within an area in the health sciences (Doyle et al., 2020).

Transcripts were read through immersively a first time to gain initial impressions and to make brief notes, and a second time using open coding to capture key ideas into codes that began to emerge inductively and naturalistically from portions of text (Elo & Kyngäs, 2008). Finally, the team analyzed transcripts to clarify and capture codes referencing more than one key idea, thus collapsing initial codes into categories, and nesting codes beneath these categories into subcategories as appropriate. Categories were then collapsed into overarching themes. Transcripts were entered into Dedoose (Version 8.3.45) for data management. The research team met several times to review and discuss findings in an iterative way. Field notes and memos were included in this review process to enhance reflexivity, and an audit trail of digital and hard copy data and notes was compiled throughout. Data analysis occurred concurrently with data review in an inductive, cyclical process, concordant with content analysis (Hsieh & Shannon, 2005) and QD (Kim et al., 2017). To ensure qualitative trustworthiness and validity, Standards for Reporting Qualitative Research (O’Brien et al., 2014) were adhered to throughout.

## Results

Eleven participants from a variety of professional disciplines, ages 25–61, 10 female and one male, participated in three focus groups (four, four, and three participants, respectively). Six participants were Registered Nurses (RNs), one a Licensed Clinical Social Worker (LCSW), and four others had extensive experience in geriatric care management with certification in care coordination. The primary workplaces for participants were the in-home community setting (five participants), an outpatient clinic (three participants), the inpatient setting (two participants), and an adult foster home (one participant).

Findings from the focus groups evolved into two primary themes: (a) *Perceptions of optimal dementia assessment practices*, and (b) *Usefulness and feasibility of the READyR Program.* The first theme, regarding optimal assessment, further distilled into two distinct categories with further content analysis: *barriers* and *facilitators* to optimal assessment. The second theme, usefulness and feasibility of READyR, similarly distilled into: READyR data *advantages* and *disadvantages.*

### Perceptions of Optimal Assessment Practices: Barriers

Dementia care coordinators expressed multiple issues impeding the ideal assessment of the needs of persons living with dementia and care partner. Some of the barriers to an ideal assessment included: complex care issues of the person living with dementia, such as degree of cognitive impairment; family skepticism regarding professional assessment or lack of readiness; assessment within busy or difficult-to-tolerate environments. Other more nuanced and complicated assessment barriers were also discussed and included: proxy reporters and family issues, such as *Family Relationship Strain*, *Negating Personhood*, or *Incongruence* between the client’s needs and perceptions of needs. All of the following exemplars have been edited for clarity.

Multiple varieties of *Family Relationship Strain* were expressed by participants. Strains encompassed by family situations wherein members were not on the same page (and could not seem to resolve conflicts) impeded the best interests of the person living with dementia and/or their care partner:

I’ve had one (case with) multiple family members that have gone back and forth about who—if they will or they won’t be the guardians and who should do that—to the point where a couple of the brothers are no longer speaking to each other because of the whole family dynamic. And finally, after all was said and done, the family members *all* exited the plan. (Focus Group 3; participant 4)

I’d say the hardest part of assessments that tend to go poorly is when it’s feuding families…. Step kids don’t like mom or dad, both of them have a cognitive issue, and if they are—if they got married later in life, the kids don’t like each other, the kids don’t like the opposite person, and it’s a lot. Very tangled. (Focus Group 2, participant 2)

Focus group participants mentioned *Negating Personhood* as an almost unconscious or reflexive stance taken by family members accustomed to assuming care of the person living with dementia. Care coordinators perceived that this often—advertently or inadvertently—impeded accurate assessment:

Sometimes I’ve done an assessment by myself and the family will answer every question for the client, and then you feel as if you don’t have… they’re not doing it on purpose, but they’re just so used to well, “I know Mom can’t answer that, so I’m going to answer for her,” and speaking for Mom. And you can’t really get an accurate picture of what’s happening. (Focus Group 2, participant 1)

(Client’s) sister was there and she turned to her sister and would say, “What do you think?” And first, her sister would express support, and then she would start challenging everything we shared. And I could just see our client was absolutely paralyzed. Because in her cognitive impairment, she was questioning herself—whether she could make those decisions. And then she was turning to her one support person who professed support but really was negating every single thing her sister was trying to decide. So, that family support, or lack of it, shows up in many different ways. (Focus Group 1, participant 3)

Not infrequently, care coordinators voiced *Incongruence* between a family member or care team member’s assessment of what was seen in the best interest of the client living with dementia (e.g., a need for the person living with dementia to stop driving; increasing need for formal care, etc.) and the client’s assessment of their own needs. Focus group participants discussed how this incongruence could cause stalemates in assessment and care planning, leading to more drastic measures to ensure safety:

That’s where we get into some of our hardest work, when patients are really convinced they are still able to make decisions. And they disagree with family members. And we have to pursue a guardianship or legal channels to enact against a patient—(against) what a patient is saying. (Focus Group 1, participant 3)

Although participants were quick acknowledge the importance of family to the assessment process, the barriers that care coordinators face to gaining an optimal assessment of care needs reflect difficulties in first understanding the care situation objectively, without the family members’ interpretation or appraisal of the needs or values of the person living with dementia. On the other hand, an inability to find common ground with the person living with dementia, and be on the same page about the needs assessment, diminishes the assessment process.

### Perceptions of Optimal Assessment Practices: Facilitators

Conversely, focus group participants discussed scenarios that facilitate optimal assessment. These scenarios fell into complementary subcategories—*Comprehensive Assessments* and *Tailored Assessments and Care.*

Care coordinators seemed to always be striving for *Comprehensive Assessments.* This revealed their expertise and creativity in gathering all information possible to create a complete picture of dementia care needs.

The assessment is pretty comprehensive. It takes a couple of hours to do. From past medical history, past surgeries; doctors, past doctors; family history, likes, dislikes; how they spent their life; falls, interventions, medications they’re currently on…. It’s quite a list. We have a home safety assessment; we do actual safety counseling. We have the geriatric depression screen. We have a variety of cognitive scores that we can utilize if that has not been done in a recent medical visit. We do a legal screen. We do financial, social—we kind of get a social history and background. And then we have a variety of other tools we can grab if need be. We do a falls assessment pretty much on everybody. But if there were skin issues … you know, I carry with me a compendium that’s listed in our assessment tool, a variety of other assessments. If there’s suicidal ideation, we put in a suicidal assessment. (Focus Group 1, participant 3)

At the same time, dynamically altering assessment strategies in real time to capture an evolving individual and care dyad’s picture, in vivo, was seen as critical to optimal assessment. This created *Tailored Assessments and Care:*

I question how did they spend their time before all of this as a couple? Family background, how did she spend her day, how did he spend his day? How much time did they spend together? Because couples are very different and unique in their marriages. So, how did that play out before these issues are starting to arise, and where is the family in this process? (Focus Group 1, participant 1)

I’ll be, “Can you show me your bedroom?” and then we talk through: “Well, how do you get ready in the morning, what’s your routine?” And that’s usually when I can get more information, more accurate information. (Focus Group 2, participant 2)

We start with an ADL assessment. So, what are those activities that they are able to do? What is it that his (care partner) is helping him with? And then, doing the caregiver burden scale as well, just to see how much it is weighing on (them). (Focus Group 2, participant 2)

I’m tying so much into my assessment, which is more, “What did you do for a living?” “What kinds of things do you enjoy doing?” And, “What’s important to you? What are your goals?” Just trying to get to know the person so that when you are communicating you are involving them. So … how can you emulate certain things? Like, if they can’t use power tools anymore, what might be something that you could show them that, “Hey, you’re still creative. You can do this. And here’s an option for you.” (Focus Group 3, participant 2)

Overall these findings indicate that getting to know the person living with dementia and their care partner better facilitates an optimal needs assessment. Through these exemplars, it is clear that care coordinators use whatever data are available to them, but tailor the assessment process in order to gain the most in-depth picture of the care situation, and current care needs. An optimal assessment process is thus one that allows the care coordinator to know the person living with dementia and their family well enough to individualize care.

### Usefulness and Feasibility of the READyR Program: Data Advantages

The objective data from READyR were seen by focus group participants as an advantage, in general, to a dementia care needs assessment. Data regarding patterns of driving, medication-taking behavior, sleep, weight, physical activity, and time out of the home were all seen as advantages that could, for example, signal safety issues, independence in daily routines, and potential strains for the care partner such as disturbed sleep. The fine-grained, longitudinal changes in these data over time were also seen by participants as a novel way of *Supporting Care Decisions and Values*, including the subcategory *Entry into Delicate Topics.*

Care coordinators recognized the potential for READyR to help in reflecting behavioral patterns back to persons living with dementia and care partners, so they could see this was not (or was) *Supporting Care Decisions and Values* they had previously identified. These values included burden, safety, autonomy, or social relationships.

The ability to see when the caregiver is going to sleep and how much they’re walking around and how much they’re spending time in the other room, including the bathroom, can kind of clue you in before you do a needs assessment that caregiver burden or stress is going to be an issue. Because sometimes when you ask caregivers how they’re doing or if they’re under stress, they’ll say, “Oh, I’m fine. I’m fine. Everything’s fine.” They want to stay in their home as long as possible. (Focus Group 1, participant 2)

Just seeing how many hours per day they’re spending in the home together could clue you in that there’s more caregiving going on. What goes through my mind is, the number of caregiving hours increased today—or at least the amount of time they spent together per day increased from 11 to 17 hours per day…. It would be a good way to bring it up with them to think about future caregiving planning. (Focus Group 1, participant 2)

In this way, participants felt READyR could generate objective, novel, and noninvasive (i.e., no person spending time observing families in the home, and no audio or video captured) data with the potential to inform difficult discussions regarding potentially increasing care needs. With READyR data in hand, care team members would be provided *Entry into Delicate Topics:* a conversation initiation tool that could be revisited and scaffolded upon over time to assist dementia care dyads in accepting, for instance, increasing needs for help in the home.

It could give you some good starting points for conversations—caregiver strain, caregiver burnout, or maybe not having any respite time or time for (herself) built in. Maybe that’s something that prompts the conversation to say, “You know, it looks like you’re spending a lot of time doing the caregiving. Let’s talk about you for a minute. What are you—where are you at? What do you put in place to help yourself with this?” (Focus Group 3, participant 1)

Well, would you rather have these sensors in your home that aren’t taking any video or audio recordings, or would you rather have a caregiver in your home for 12 hours so that we understand what’s happening? (Focus Group 2, participant 2)

### Usefulness and Feasibility of the READyR Program: Data Disadvantages

Though prompted repeatedly, care coordinators expressed few perceived disadvantages to the remote digital data produced by READyR. One disadvantage identified was that it seemed likely some families would view sensor technology as invasive. Although the technology platform does not capture any audio or video data, participants noted that it could alter the home in some way that might be uncomfortable for residents living with dementia and the spouses or other care partners, especially those who are not accustomed to technology. Focus group participants also discussed the READyR Program’s inherent limitations when it comes to capturing the full care situation in the home, as the following exemplar illustrates. Yet in general, disadvantages mentioned were scarce and no subcategories emerged.

It feels invasive, even though it’s not necessarily video, it’s something in their home, they’re not tech-savvy and it will feel like—it’ll just feel uncomfortable for them. (Focus Group 2, participant 1)

Clearly these are data that are for the two people, and how much are we missing from support from other people coming into the home? I’m not sure that these data can accurately capture that. (Focus Group 2, participant 3)

## Discussion

Assessing the everyday care needs of persons living with dementia is the fundamental first step to identifying care supports and services that can promote well-being in dementia. The READyR Program defines a care need as the mismatch between what is most important to the person living with dementia (i.e., autonomy, safety, avoiding burden on family, or social relations) and how patterns of daily activities and experiences may or may not uphold the particular care value (e.g., independent morning routines, taking medications on time, not disturbing each other’s sleep, and having visitors). The development process for the READyR Program has yielded a values-based in-home needs assessment tool that incorporates objective data and minimally obtrusive remote monitoring. Feedback from focus groups with care coordinators indicates that the READyR Program has multiple advantages and potential uses for gaining a continuous and ecologically valid dementia care needs assessment in the community setting.

Our dementia care needs assessment program expands upon existing care planning and interventions with technology in several ways. First, READyR assesses the care values of the person living with dementia, or what is most important to the person living with dementia. This information is critical to knowing the person living with dementia, and has been identified as an essential element of planning for future care (Lord et al., 2020; Read et al., 2021). Practically speaking, knowing what is most important to the person living with dementia helps to focus the dementia care needs assessment on the areas most central to each individual’s well-being, rather than attempting to give equal attention to an extensive and often overwhelming inventory of potential care needs. However, the window of opportunity for assessing the care values of the person living with dementia closes at sometime in the moderate to severe stage of dementia, when reliably communicating about what is important becomes too difficult. Second, digital activity metrics taken from the home environment are ecologically valid, ongoing, and are highly sensitive to change, which is important to gain a clearer window into the dyad’s routines within the home over time, and to detect the efficacy of interventions. Third, in the development of READyR, the use of technology for collecting objective data and assessment of care needs was guided by the READyR framework and the goal of assessing in-home activity patterns, rather than letting the technology dictate the intervention design.

In the focus groups, care coordinators identified barriers to current assessment practices that highlight the need for more than a single assessment in the home, and ideally including objective data. Family relationship strain can divert the focus of a dementia care needs assessment to the interpersonal dynamics in the family. Strain in the relationship also negatively affects the family caregiver’s subjective appraisal of the values of the person living with dementia (Miller et al., 2018, 2019; Reamy et al., 2011), which ultimately impairs the ability to assess care needs. One clear advantage of the READyR Program is that data from sensors have the capacity to provide new objective insights into safety, function, and routines in the home.

### Limitations

Overall, the results from the focus groups in this study, as well as other studies of dementia care planning, suggest a great need for a values-based in-home needs assessment program such as the one developed here. Nevertheless, this study is not without limitations. Self-selection of focus group participants who were interested likely increased the amount of positive feedback on the advantages of the READyR Program, and likely decreased the amount of feedback on disadvantages. There is a possibility that the nature of recording focus groups over videoconference also limited the participants to those with better access to internet-connected devices and an interest in using technology in their profession, which could have also biased results in favor of READyR’s use of technology in the homes of persons living with dementia.

There are other limitations of the READyR Program that were not captured in focus groups, but are likely to emerge as the pilot testing gets underway. As with all technology-based interventions, technology failures or disruptions in data transmission could threaten the ability of the study team to get accurate and timely data. There are also potential challenges in communicating about the data to families, given the range of health and digital literacy and the contexts of dementia and older adults with potential sensory deficits. READyR was designed to complement, not replace, comprehensive evaluations and in-person needs assessments within the home. Pilot testing will elucidate the extent to which remote assessments will be feasible, acceptable, and efficacious among persons living with dementia and their care partners. Finally, READyR is currently most appropriate for persons with early-stage dementia due to the need to assess care values directly, yet care planning interventions are still important at all stages of the disease process.

### Future Directions

Ultimately, the goal is for READyR to be integrated with other successful care planning programs and health care delivery systems as a seamless, dynamically tailored enhancement to dementia assessment and care. The next step toward this goal is to determine the feasibility, acceptability, and efficacy of the READyR Program for identifying care-related needs of persons living with dementia and improving the well-being of care dyads through pilot testing and a full-scale clinical trial. With further refinement and testing, the READyR Program may be well suited to a pragmatic trial that embeds the remote monitoring into a clinical care coordination framework. Continuity of care could dramatically improve with access to information about how well the family care dyad is able to sustain the in-home routines that are most important to meeting care needs in the home. By working from a values-based perspective, READyR can capture personalized areas of need, which, in turn, can help to inform the development of a plan to address these unmet needs. As dementia progresses, routines and functional capacity will change and decline, and future studies will need to examine the READyR Program longitudinally to optimize the process for assessing needs throughout the course of dementia care.

## Conclusion

Despite the broad availability of tools and services for future care planning and preparedness, only a small proportion of the older population in general uses them (Sörensen et al., 2017). The READyR Program has the potential to help family members as well as care coordinators and providers gain insight into the values-based everyday care needs of persons with early-stage dementia. Given the broad reach and integration of technologies in the homes of older adults, READyR is well-positioned to enhance care planning and aging-in-place by pairing more continuous and ecologically valid assessment of in-home activities with better understanding of what is most important to the person living with dementia.

## Supplementary Material

igac006_suppl_Supplementary_Material
