# Supplementary material for: The Remote Assessment and Dynamic Response Program: Development of an In-Home Dementia-Related Care Needs Assessment to Improve Well-Being
Source: Innov Aging. 2022 Feb 7;6(2):igac006. doi: 10.1093/geroni/igac006 (PMC8985764; doi:10.1093/geroni/igac006)
Supplement: igac006_suppl_Supplementary_Material [file igac006_suppl_supplementary_material.docx]

**READyR Semi-structured Case Manager Focus Group Interview Guide**

| 1. Talk about your current process for assessing dementia-related care needs among patients who have been diagnosed with dementia, and their spouses or other close family members. |
| --- |
| 1. What types of information about the patient’s activities and home environment are you usually able to incorporate into your assessment of dementia-related care needs? Specifically, what information do you tend to gather about the autonomy or independence of the patient, about the burden/stress on the caregiver, about safety, and about their social life? |
| 1. In what situations is it part of your role to assess dementia-related care needs?    1. Can you give me an actual example of a time when things went well and why?    2. Can you offer an example of when things were less than optimal and why? |
| - *Break in questions to present case study and sensor data -*   I am about to present to you with some data about a couple living with dementia. Please keep in mind that this is actual data that is gathered continuously and unobtrusively in the home from a variety of sensors. You have the option to take notes about anything that occurs to you about the data as I am presenting it.   - *Read case study and give sensor data presentation -* |
| 1. What are your initial thoughts about the information–please share anything and everything that comes to mind about the usefulness of the data? |
| 1. If you had this additional information from sensors in the homes of patients with dementia available to you, how might you use it in a dementia-care needs assessment?    1. How feasible would it be for you to incorporate this type of data? |
| 1. What do you think this objective data could add to your picture of current and future care needs?    1. From what you saw, what data would you be most interested in having available to you?    2. What data, if any, did you not see that you would like to have available to you when conducting an assessment of dementia-related care needs? |
| 1. If a patient with dementia valued their autonomy first and foremost, what types of data do you use already or would you like to use to help you understand their needs related to supporting autonomy? |
| 1. If a patient with dementia valued not being a burden on their spouse for their care, what types of data do you use already or would you like to use to help you understand their needs related to helping take the burden off their spouse? |
| 1. If a patient with dementia valued their safety first and foremost, what types of data do you use already or would you like to use to help you understand their needs related to supporting safety? |
| 1. If a patient with dementia valued their social relations first and foremost, what types of data do you use already or would you like to use to understand their needs related to supporting social relations? |
| 1. Do you have any other thoughts about what information would be most useful to you when you are assessing dementia-related care needs? |
